# Dying feet in ICU: why might extracorporeal membrane oxygenation machines cause necrotic feet?

**DOI:** 10.1186/1757-1146-6-S1-O26

**Published:** 2013-05-31

**Authors:** Helen V Martin, Peter A Lazzarini, Ewan M Kinnear, Lynette Munck, John Fraser

**Affiliations:** 1 Critical Care Research Group, The Prince Charles Hospital, Queensland Health, Brisbane, Queensland, 4032, Australia; 2 Department of Podiatry, Metro North Hospital & Health Service, Queensland Health, Brisbane, Queensland, 4032, Australia; 3 Allied Health Research Collaborative, Metro North Hospital & Health Service, Queensland Health, Brisbane, Queensland, 4032, Australia; 4 School of Clinical Sciences, Queensland University of Technology, Brisbane, Queensland, 4059, Australia

## Background

Extracorporeal membrane oxygenation (ECMO) is used for severe lung and/or heart failure in intensive care units (ICU). The Prince Charles Hospital (TPCH) has one of the largest ECMO units in Australia. Its use rapidly increased during the H1N1 (“swine flu”) pandemic and an increase in pedal complications resulted. The relationship between ECMO and pedal complications has been described, particularly in children, though no strong data exists. This paper presents a case series of foot complications in patients having received ECMO treatment**.**

## Methods

We present nine cases of severe foot complications resulting from patients receiving ECMO treatment at TPCH in 2009–2012.

## Results

Case ages ranged from 16 - 58 years and three were male. Six cases had an unremarkable medical history prior to H1N1 or H1N2 infection, one had Cardiomyopathy, one had received a lung transplant, and one had multi-organ failure post-sepsis. Common medications prescribed included vasopressors, antibiotics, and sedatives. All cases showed signs of markedly impaired peripheral perfusion whilst on ECMO and seven developed increasing areas of foot necrosis. Outcomes include two bilateral below knee amputations, two multiple digital amputations, one Reflex Sympathetic Dystrophy Syndrome, three pressure injuries, and three deaths.

## Conclusion

Necrosis of the feet appears to occur more readily in younger people requiring ECMO treatment than others in ICU. The authors are conducting further studies to investigate associations between particular infections, medical history, medications, or machine techniques and severe foot complications. Some of these early results will also be presented at this conference.

